# Saratoga Springs

**Published:** 1876-09

**Authors:** 


### *SARATOGA SPRINGS.*

A good many readers of HALL'S JOURNAL OF HEALTH are visiting Saratoga this season. It is a wise thing for hundreds of them to do, if they will only use wisely the opportunity for recuperation which will thus be afforded. But such as seek restoration from disease, or, being well, are vigilant to continue so, may like to receive a hint or two.

The mineral spring waters are used by everybody. Some of the waters are tonic, some diuretic, some cathartic. It is evident that medicinal agents like these, even though cunningly compounded in nature's own laboratory,should not be thoughtlessly and indiscriminately imbibed. If we should deem it wise to enter a doctor's shop and swallow pills and powders recklessly, we might also manifest wisdom in carelessly partaking of the potent medicated solutions of Saratoga. But intelligence and experience are safer guides than caprice. Our friends will do well to consult some educated physician at the Springs before indulging largely in the sparkling fountains of that popular resort. The Drs. Strong, father and son, can tell you what water you may drink and how much, and we commend our readers to their intelligent consideration. They have had long experience with the waters, as well as in the treatment of diseases by all known medical and surgical appliances, and are constantly receiving patients consigned by physicians residing far away. Besides, they are the proprietors of a delightful home for invalids or for well folks, and entertain some thousands each year. Their table is abundantly supplied with all the good things in the market, and a true home-feeling is always acknowledged among the guests. We have visited them, and have been made to feel—as all guests seem to—that instead of being lost in the loneliness of a crowd, as we have when a guest of some one of the great Saratoga hotels, we are, at the Drs. Strong's, really a part of one large but friendly family circle, among a class of thoughtful and cultivated persons from whom much may be learned, and whose acquaintance we shall be glad to continue in after years. We say, then, to our readers who visit Saratoga, that they will do wisely to consult the Drs. Strong before drinking the waters of the Springs, and that they will thank us for recommending the widely-known remedial institute of these gentlemen as their abiding-place at any season of the year, whether seeking a home only, or, in addition, requiring the advice or aid of medical knowledge and skill. These were the sentiments of the late Dr. Hall, as our readers are aware, and they are no less our own.

It is the fashion to visit this popular watering-place in the summer months. During the heated term inhabitants of thickly settled localities migrate to other quarters, and derive benefit from the change. This is all very wise. But there are invalids, and worn-out people, and sufferers from disease, who can not get well unless they surround themselves with appropriate health-restoring conditions, and who may wait too long if they wait till the fashionable summer flitting-time. To this class we have a word to say.

Henry Clay once remarked that he "would rather be right than be President." So it is better for you to be well than to be fashionable. If you are sick, with any chronic ailment, the chances are that you will not recover until you place yourself under appropriate conditions for recovery. Perhaps you need rest and freedom from household care. It may be that your diet is not well suited to your condition ; that the atmosphere of your dwelling is not the atmosphere which you should dwell in while seeking health. Possibly there exists some brain excitant, some cause of worry, some nerve irritant, which renders recovery impossible. If so, you need a change. Your duty is, *to make a business of getting well.* If you can not do this at home—and only the very rich can, if, indeed, it is possible for them—then you must seek a locality and surroundings and appliances and skill adapted to your needs, and devote the necessary period to intelligent efforts for recovery.

Now, it seems to us that there is no better place to get well in than the Remedial Institute of the Drs. Strong. It seems to us, also, that the autumn and winter are the best seasons for the purpose. There is then no crowd, no excitement, no tendency to late hours, undue exertion or dissipation. The temperature is better adapted to recovery ; the atmosphere of the region is less humid and of higher tonic power. Being inland, and surrounded by mountains, there are no sudden changes and no severe winds. The air is said to be rich in ozone, and, therefore, free from malaria. The temperature being comparatively equable, the locality is well adapted to such as suffer from the severity of the northern winter. The town of Saratoga has 10,000 permanent citizens, and supplies cultivated society and all the social pleasures which attend refinement and culture.

The Remedial Institute is amply provided with all modern appliances for the cure of disease. There are the Turkish, Russian, Sulphur, Hydropathic, and Electro-Thermal Baths, the Equalizer Vacuum Treatment, Laryngoscope, Oxygen Gas, Faradaic and Galvanic Electricity, Health Lift, Calisthenics, and the like, together with the vast materia medica of the educated physician.

The Institution has the appointments, appurtenances, and elegance of a first-class modern hotel. Its ample halls and gymnasium are thoroughly warmed by steam and well ventilated. Its extensive piazzas afford opportunities for exercise at all times. No one can visit it without being convinced of its desirableness as a winter home.

With such a climate, springs, location, and an Institution of such complete curative appliances, fully indorsed by the medical profession, especially fitted for the home comfort and treatment of invalids, and the skill attained by an experience of more than twenty years in the treatment of Nervous, Lung, Female, and all forms of Chronic diseases, the invalid will find ample reasons for preferring the Saratoga Remedial Institute as a health resort.

